# Simultaneous Presentation of Brainstem and Cerebellar Posterior Reversible Encephalopathy Syndrome With Acute Cerebral Infarction

**DOI:** 10.7759/cureus.34843

**Published:** 2023-02-10

**Authors:** Hajime Maruyama, Hirohisa Fujikawa, Ryuichi Takimiya, Hiroki Sato

**Affiliations:** 1 Department of Internal Medicine, Suwa Central Hospital, Nagano, JPN; 2 Department of Internal Medicine, Fujimi-Kogen Hospital, Nagano, JPN; 3 Department of Medical Education Studies, International Research Center for Medical Education, Graduate School of Medicine, The University of Tokyo, Tokyo, JPN; 4 Department of Diagnostic and Generalist Medicine, Dokkyo Medical University, Tochigi, JPN; 5 Department of Neurosurgery, Suwa Central Hospital, Nagano, JPN

**Keywords:** hypertension, antiplatelet therapy, blood pressure management, acute cerebral infarction, posterior reversible encephalopathy syndrome (pres)

## Abstract

Posterior reversible encephalopathy syndrome (PRES) and cerebral infarction are both caused by hypertension, but they rarely occur together. If they do coexist, the selection of a management strategy is difficult because of their pathologic differences. Here, we present an uncommon case of brainstem and cerebellar PRES combined with acute lacunar infarction. For this patient, we used an aggressive blood pressure-lowering regimen during the acute phase of his condition. Once the cerebral edema caused by PRES began to improve, antiplatelet therapy was initiated. The treatment was ultimately successful, and the patient was discharged home. A return to work is now planned. Given the rarity of this combination of conditions and a lack of published evidence for management, our report will contribute to the literature concerning the treatment for this combination of conditions.

## Introduction

Hypertension is a major public health issue and a risk factor for cardiovascular and cerebrovascular diseases throughout the world [1]. Although both cerebral infarction and posterior reversible encephalopathy syndrome (PRES) are caused by poorly controlled hypertension, the two conditions are completely different and rarely occur together. If both develop in the same patient, management becomes challenging [2]. Here, we present a successfully treated case of the brainstem and cerebellar PRES coexisting with cerebral infarction.

## Case presentation

A 53-year-old man with a history of untreated hypertension presented with a lean to the left and a headache. Several hours after the onset of the symptoms, the patient visited our hospital. On examination, his blood pressure and heart rate were 214/145 mmHg and 84 bpm, respectively. A neurologic examination revealed dysarthria; weakness of the left quadriceps and hamstrings, with manual muscle testing of 4/5; and bilateral cerebellar ataxia.

**Figure 1:**
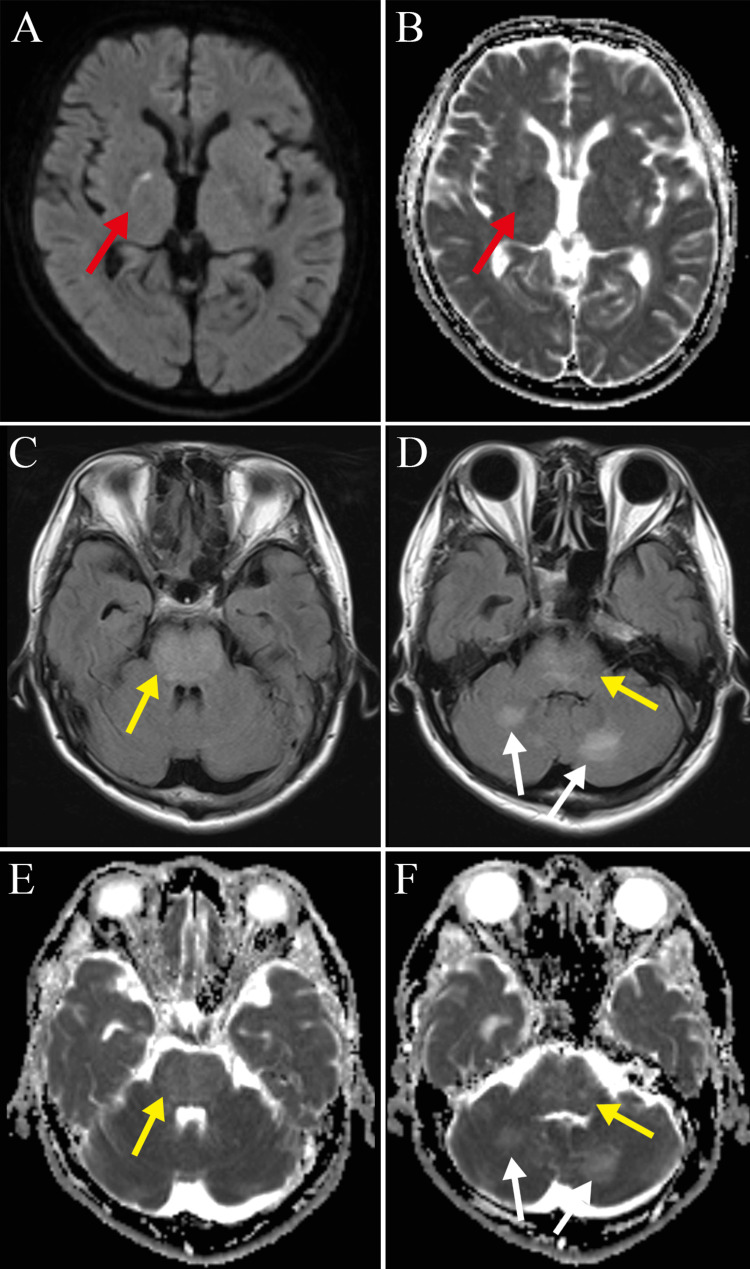
Cranial magnetic resonance imaging at admission (A,B) Diffusion-weighted imaging (DWI) shows a high signal, and the apparent diffusion coefficient (ADC) map shows a low signal extending from the right centrum semiovale to the posterior limb of the internal capsule (red arrows). (C,D) Fluid-attenuated inversion recovery (FLAIR) images and (E,F) the ADC map both show a high signal in the pons (yellow arrows) and bilateral cerebellar hemispheres (white arrows).

Blood tests showed high low-density lipoprotein cholesterol (201 mg/dL) and mildly elevated serum creatinine (1.13 mg/dL). Liver function, fasting blood glucose, and hemoglobin A1c tests were all within the normal range. Cranial magnetic resonance imaging (MRI) produced two significant findings. First, a lesion extending from the right centrum semiovale to the posterior limb of the internal capsule showed a high diffusion-weighted imaging (DWI) signal and a low apparent diffusion coefficient (ADC) signal, indicating an acute-phase lacunar infarction (Figure 1, Panels A and B). Second, fluid-attenuated inversion recovery (FLAIR) images and ADC revealed extensive hyperintense lesions in the brainstem and bilateral cerebellar hemispheres, indicating PRES (Figure 1, Panels C-F). The patient was admitted for a suspected lacunar infarction coexisting with PRES.

**Figure 2:**
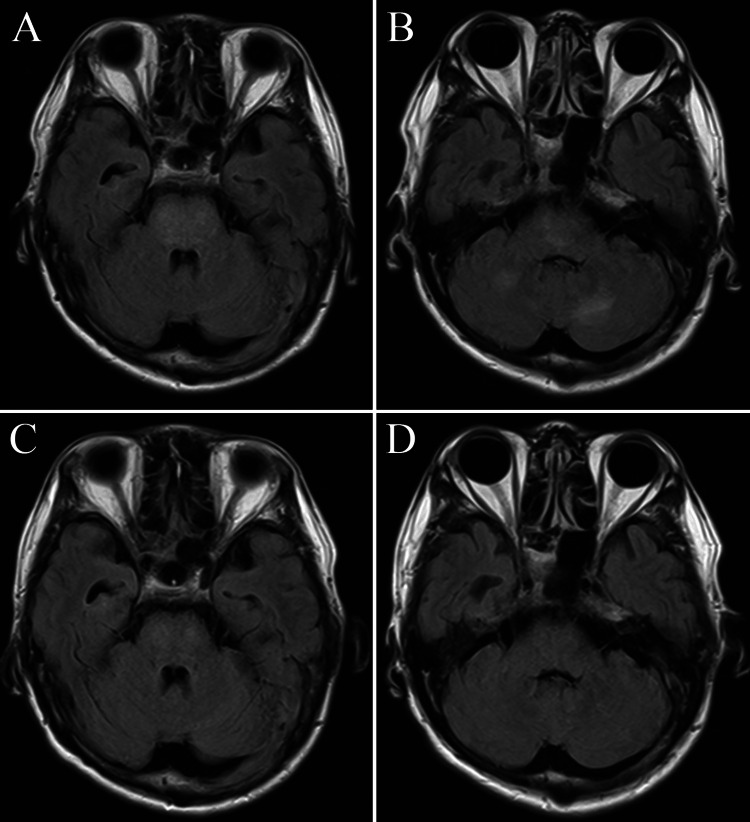
Magnetic resonance imaging of the head during hospitalization (A,B) Cranial magnetic resonance imaging on day 4 of admission. Fluid-attenuated inversion recovery (FLAIR) images show lightening of the high signal in the pons and cerebellum. (C,D) Cranial magnetic resonance imaging on day 21 of admission. On FLAIR images, the high-signal lesions have disappeared.

Upon admission, intravenous nicardipine was administered to lower the patient’s systolic blood pressure to less than 140 mmHg. On day 11 of admission, the patient’s antihypertensive therapy was switched from intravenous nicardipine to oral nifedipine with azilsartan. Considering the risk of hemorrhage from the PRES lesions, antiplatelet agents were not started immediately. On day 4 of admission, FLAIR images on MRI revealed a reduction in the high signal in the brainstem and cerebellum that had suggested PRES (Figure 2, Panels A and B); antiplatelet therapy was therefore initiated. For secondary prevention, rosuvastatin was administered from day 1 of admission.

On day 21 of admission, cranial MRI showed clear improvement in the bilateral cerebellar hemispheric and brainstem lesions, a course consistent with PRES (Figure 2, Panels C and D). The patient’s final management regimen consisted of nifedipine 20 mg, azilsartan 40 mg, aspirin 100 mg, and rosuvastatin 2.5 mg daily, which maintained the patient in stable condition, without recurrence of the PRES and infarction or development of any intracranial hemorrhage. After an intensive course of rehabilitation, the patient was discharged home. Two months after discharge from our hospital, the patient is doing well, with no recurrence. He is planning a gradual return to work.

## Discussion

Reports of the brainstem and cerebellar PRES occurring together with cerebral infarction are few, and only a little evidence for a management strategy has been developed. The present case posits a management approach for this rare condition.

PRES is characterized by clinical symptoms such as headache, altered mental status, seizures, and visual abnormalities. Although details of its pathogenesis remain unknown, the precipitating factor is believed to be vasogenic edema. Causes of vasogenic edema include impaired cerebral autoregulation from severe hypertension, vascular endothelial dysfunction from sepsis, bone marrow transplantation and chemotherapy, and increased permeability of the blood-brain barrier related to immune system activation [3]. On imaging, PRES generally presents as subcortical and cortical edema of the cerebral hemispheres, with the parietal and occipital lobes most frequently being involved. However, deep structures and other areas can also be affected. A high signal on T2-weighted MRI and FLAIR sequences is more sensitive for detecting PRES lesions [4]. The hallmark vasogenic edema in PRES presents with a hypo- or iso-intense signal on DWI and high ADC values. In contrast, the main pathologic change in early cerebral infarction is cytotoxic edema, which presents with marked hyperintensity on DWI and low ADC values [5].

PRES generally has a good prognosis: it is frequently fully reversible within a period of days to weeks if treated early and adequately (i.e., with the removal of the triggering factor and appropriate blood pressure control) [6]. However, PRES can also result in death and permanent neurologic disability [7]. Death can occur as a result of progressive cerebral edema, intracerebral hemorrhage, or underlying disease [8]. Cerebral hemorrhage is reported to occur in about 15% of PRES cases [9]. Microbleeds are reported to occur even more often [10]. Early diagnosis and management of bleeding risks are therefore vital.

PRES lesion sites typically include the occipital lobe (98.7%), posterior frontal lobe (78.9%), and temporal lobe (68.4%). Atypical sites, accounting for approximately 4% of lesions, include the cerebellum, basal ganglia, brainstem, and deep white matter [5]. Finding PRES lesions in the brainstem, cerebellum, and basal ganglia without parietal or occipital involvement, as in this case, is rare [5]. However, it is important to note that brainstem and cerebellar PRES can cause acute posterior fossa circulation failure and thus obstructive hydrocephalus [11]. Brainstem lesions are considered a poor prognostic factor [12].

In a previous report with a summary of 16 cases of brainstem PRES, the authors stated that no clear explanation for the mechanism that leads to PRES lesions being more prominent in the brainstem than in the occipital lobes had been presented [13]. However, two hypotheses have been proposed. The first suggests that the distal part of the vertebrobasilar artery system (the parieto-occipital region) might be preserved because of extensive fluid leakage in the brainstem. The second hypothesizes that the preservation of this region relates to its richness in sympathetic nerves and blood flow from the anterior circulation via the posterior communicating artery [13].

The combination of cerebral infarction and PRES is rare, and Table 1 summarizes the few reported cases [2,14-16]. Only one prior case of cerebral infarction combined with brainstem and cerebellar PRES has been published, and the PRES lesion, in that case, was confined to the infratentorial region, as in our patient [14]. In general, the brainstem is not vulnerable to hypertensive conditions, but an unexpectedly rapid elevation of blood pressure might cause a brainstem lesion. In our case, the patient had a history of hypertension. The onset of his cerebral infarction was assumed to cause a sudden elevation in blood pressure, which was thought to lead to the development of PRES.

**Table 1:** Characteristics of patients with posterior reversible encephalopathy syndrome coexisting with cerebral infarction HTN: Hypertension; PRES: Posterior reversible encephalopathy syndrome.

Author (year) [Ref.]	Patients			Site of lesions		Treatment
	Age (years)	Sex	Past medical history	PRES	Cerebral infarction	
							Kamada et al. (2014) [14]	46	Male	HTN	Brainstem, cerebellum	Basal ganglia, posterior limb of the internal capsule	Antihypertensive medication from day 1 of admission (nicardipine -> combination of five drugs); no antithrombotic therapy
Kazahari et al. (2018) [15]	48	Male	HTN	White matter of the fronto-temporoparietal lobes, pons	Corona radiata	Antihypertensive medication from day 1 of admission (nicardipine -> amlodipine); aspirin, atorvastatin (start date unknown)
Liu and Zhang (2020) [2]	36	Male	None	Pons, hippocampus	Thalamus	Nifedipine, aspirin, and atorvastatin from day 1 of admission
Suzuki et al. (2022) [16]	66	Male	None	Cerebral cortex, thalamus	Medulla oblongata	Nicardipine only on days 1-2 of admission; anticoagulants from day 2 of admission (argatroban -> edoxaban)

No clear management strategy for brainstem and cerebellar PRES combined with cerebral infarction has been established. Individually, these conditions have different management strategies. Moreover, the treatments for hypertension and the timing for introducing antithrombotic drugs are controversial when the two conditions are combined. In PRES of the brainstem and cerebellum, bleeding at the lesion site can be fatal; therefore, while choosing the treatment, particular consideration must be given to the risk of bleeding. In our case, the choice was to begin with antihypertensive therapy to treat the PRES. Antithrombotic therapy, a treatment for cerebral infarction, was not initially used. Antithrombotic therapy was initiated after improvement of the cerebellar and brainstem edema was demonstrated on MRI [17]. It has been reported that antihypertensive therapy for acute lacunar infarction does not worsen the functional prognosis [18]. A management strategy that prioritizes blood pressure control to reduce bleeding risk could therefore be appropriate. Furthermore, antithrombotic therapy is believed to increase bleeding in PRES [9]. In reports of PRES with edema and intraparenchymal hemorrhage, the hemorrhage was reported to have occurred in the same location as the edema or within a similar vascular distribution in all patients; hemorrhage was not observed in areas without edema [17]. Thus, in the present case, we initiated antiplatelet drugs after the cerebral edema improved; however, there is still insufficient evidence in this regard, and the risks and benefits should be fully considered in shared decision-making with patients.

## Conclusions

Herein, we report a rare case of PRES coexisting with acute cerebral infarction. Our management strategy was to aggressively lower the blood pressure in the acute phase and to initiate antiplatelet therapy when the vasogenic edema of PRES abated. We hope that this report can be useful to other clinicians encountering this combination of conditions. Additional case reports and further research on this topic are required.
